# Quantitative characterization of L-citrulline as a precursor of L-arginine in rats: pharmacokinetic basis for nitric oxide-mediated effects

**DOI:** 10.3389/fphar.2026.1889835

**Published:** 2026-09-03

**Authors:** Sungmin Song, Subindra Kazi Thapa, Mahesh Upadhyay, Seonwoo Yang, Taesun Kim, Seung-Dong Yoo, Beom Soo Shin, Soyoung Shin

**Affiliations:** 1 School of Pharmacy, Sungkyunkwan University, Suwon, Gyeonggi, Republic of Korea; 2 Monash Institute of Pharmaceutical Sciences, Monash University, Melbourne, VIC, Australia; 3 Department of Pharmacy, College of Pharmacy, Wonkwang University, Iksan, Jeonbuk, Republic of Korea; 4 Australian Institute of Tropical Health and Medicine (AITHM), James Cook University, Cairns, QLD, Australia; 5 College of Pharmacy, Chung-Ang University, Seoul, Republic of Korea; 6 Department of Global Innovative Drugs, The Graduate School of Chung-Ang University, Seoul, Republic of Korea

**Keywords:** eNOS, L-arginine, L-citrulline, nitric oxide, oral bioavailability, population pharmacokinetic modeling

## Abstract

L-Arginine (ARG) is the primary substrate for nitric oxide (NO) synthesis via nitric oxide synthase (NOS), and L-citrulline (CIT) is its endogenous precursor. Although oral CIT has been reported to increase systemic ARG levels, the quantitative basis for its *in vivo* conversion to ARG and contribution to NO-mediated effects remains unclear. Therefore, this study aimed to quantitatively characterize the pharmacokinetic relationship between ARG and CIT *in vivo* and to determine whether CIT-derived ARG exposure could explain the pharmacodynamic effects observed after oral supplementation. Sprague-Dawley rats received oral ARG (500 mg/kg, n = 6), CIT (500 mg/kg, n = 6), or an equidose L-alanine control (n = 6) daily for 30 days. After 30 days of supplementation, both ARG and CIT groups exhibited significantly elevated plasma NOx levels, approximately 1.6-fold higher eNOS protein expression in the aortic arch compared with controls (p < 0.05), and reduced blood glucose concentrations. Plasma concentration–time profiles of both analytes following intravenous and oral administration of each compound were simultaneously analyzed within a single population pharmacokinetic model. The model estimated higher oral bioavailability for CIT than for ARG (F_CIT_, 82.2% vs. F_ARG_, 52.6%). It also indicated that CIT elimination was predominantly mediated by conversion to ARG (F_met_ = 0.999), resulting in greater systemic ARG exposure following oral CIT than following direct oral ARG administration. These findings provide a quantitative pharmacokinetic basis for the NO-mediated pharmacodynamic effects observed with both compounds and support the role of CIT as an effective oral precursor of ARG.

## 1 Introduction

Nitric oxide (NO) is a key signaling molecule in vascular physiology, regulating endothelial function, vascular tone, and metabolic homeostasis through endothelial nitric oxide synthase (eNOS)-mediated synthesis (Lundberg and Weitzberg, 2022). In addition to its role in vasodilation, NO is involved in glucose uptake, insulin sensitivity, and mitochondrial function (Bahadoran et al., 2020). Reduced NO bioavailability has been implicated in the pathogenesis of cardiovascular and metabolic disorders, including hypertension, atherosclerosis, and insulin resistance, making the ARG–NO pathway an important therapeutic target (Bahadoran et al., 2020; Lundberg and Weitzberg, 2022).

L-Arginine (ARG) is the sole substrate for NO synthesis by eNOS, and oral ARG supplementation has been widely investigated as a strategy to enhance NO production. However, oral ARG undergoes extensive first-pass extraction by intestinal and hepatic arginase, resulting in low and inconsistent oral bioavailability (Schwedhelm et al., 2008; Allerton et al., 2018). This pharmacokinetic limitation reduces the efficiency of oral ARG supplementation and may contribute to variability in systemic ARG exposure.

To overcome these limitations, L-citrulline (CIT) has emerged as an alternative strategy for enhancing systemic ARG availability. Unlike ARG, CIT is not a substrate for arginase and therefore avoids first-pass extraction in the intestine and liver, enabling more efficient absorption. After intestinal uptake, CIT is transported to the kidneys, where it is converted to ARG via argininosuccinate synthetase and argininosuccinate lyase. This conversion pathway provides a sustained source of systemic ARG and may support more prolonged ARG exposure than direct ARG administration (Moinard et al., 2008; Papadia et al., 2018). Consistent with this mechanistic advantage, several studies have shown that CIT supplementation increases circulating ARG levels and produces pharmacodynamic effects comparable to those of direct ARG supplementation (Schwedhelm et al., 2008; Park et al., 2023).

Although the CIT-to-ARG conversion pathway is well established, the extent to which CIT elimination contributes to ARG formation and systemic ARG exposure has not been quantitatively defined *in vivo*. In particular, no pharmacokinetic model has simultaneously characterized the disposition of both compounds with explicit quantification of the metabolic conversion fraction. The lack of quantitative *in vivo* characterization has limited the mechanistic understanding of the NO-mediated effects of ARG and CIT supplementation.

Therefore, the aim of the present study was to quantitatively characterize the *in vivo* pharmacokinetic relationship between ARG and CIT and to integrate these findings with pharmacodynamic outcomes following oral supplementation. To this end, a population pharmacokinetic model was developed to simultaneously describe ARG and CIT disposition, including endogenous synthesis and fractional conversion of CIT clearance to ARG. This approach provides a quantitative framework for linking CIT disposition to systemic ARG exposure and the resulting NO-mediated pharmacodynamic effects.

## 2 Materials and methods

### 2.1 Chemicals and reagents

L-arginine, L-citrulline, L-alanine, N-(1-naphthyl) ethylenediamine dihydrochloride (NEDD), sulfanilamide (SULF), vanadium (III) chloride (VCl_3_), and sodium nitrite were purchased from Sigma Aldrich Chemical Corp. (St. Louis, MO, USA). ^13^C_6_-ARG and D_4_-CIT were purchased from Cambridge Isotope Laboratories, Inc., (Andover, MA, USA). Acetonitrile, acetic acid, trifluoroacetic acid, and all other reagents were of analytical and high-performance liquid chromatography (HPLC) grades. All water used throughout the experiment was purified using a Milli-Q water purification system.

### 2.2 Animal studies

All animal studies were conducted in accordance with the Guidelines for the Care and Use of Laboratory Animals. The studies were approved by the Ethics Committee for the Treatment of Laboratory Animals at Wonkwang University (WKU19-13) and Chung-Ang University (CAU-202501020139). All rats were housed in plastic cages with free access to standard rat chow and water and maintained at 22 °C–24 °C with a 12 h light-dark cycle and relative humidity of 50% ± 10%.

#### 2.2.1 Repeated-dose study

Male Sprague-Dawley (SD) rats (7 weeks of age) were obtained from Hanil Laboratory Animal Center (Wanju, Korea) and acclimatized for 1 week prior to the experiment. At 8 weeks of age, rats were randomly divided into three groups (n = 6 per group): a control group receiving L-alanine as an equidose control (500 mg/kg/day), an ARG group receiving L-arginine (500 mg/kg/day), and a CIT group receiving L-citrulline (500 mg/kg/day). L-Alanine was selected as the equidose control as it is an amino acid unrelated to the ARG–NO synthesis pathway, allowing non-specific effects of amino acid administration to be distinguished from compound-specific effects. All treatments were administered once daily for 30 days by oral gavage.

Blood glucose was measured from the tail vein prior to administration on days 0, 3, 7, 15, and 30. Body mass was recorded daily prior to each administration. At day 30, urine samples were collected in metabolic cages for 12 h following drug administration. At the end of the study, animals were sacrificed, and aortic arch tissue was harvested and stored at −80 °C until further analysis.

#### 2.2.2 Single-dose study

Male SD rats (8 weeks of age) were obtained from Samtako BIO KOREA (Osan, Korea) and fasted for 12 h prior to administration. Rats were randomly assigned to four groups: intravenous ARG (250 mg/kg, n = 4), intravenous CIT (250 mg/kg, n = 4), oral ARG (500 mg/kg, n = 6), and oral CIT (500 mg/kg, n = 5). These doses were selected to ensure adequate quantification of pharmacokinetic profiles above the high endogenous baseline concentrations inherent to both ARG and CIT as endogenous amino acids. Intravenous doses were injected via the jugular vein, and oral doses were administered by oral gavage.

To determine individual baseline plasma concentrations, blood samples were collected at −2, −1, and 0 h prior to administration, and the mean of these three measurements was used as the baseline value for ARG and CIT. Blood samples were then collected at 0, 0.083, 0.25, 0.5, 0.75, 1, 1.5, 2, 3, 4, 6, 12, and 24 h after drug administration. Plasma was obtained by centrifugation at 16,000 × *g* for 10 min at 4 °C and stored at −20 °C until analysis.

### 2.3 Plasma and urinary NOx determination

Total nitrite and nitrate (NOx) concentrations were determined in plasma and urine collected at day 30. Plasma samples were diluted with water (50:50, v/v), and acetonitrile (1:1, v/v) was added to precipitate proteins. NOx was measured as the sum of NO_2_^−^ and NO_3_^−^ according to the method described by Miranda et al. (2001) (Shin et al., 2015). Briefly, 100 µL of supernatant was applied to a 96-well plate, followed by the addition of 100 µL vanadium (III) chloride (8 mg/mL in 1 M HCl) to reduce nitrate to nitrite. Griess reagents were then added sequentially: 50 µL of sulfanilamide (2%) and 50 µL of N-(1-naphthyl)ethylenediamine dihydrochloride (0.2%). After incubation at 37 °C for 30 min, absorbance was measured at 540 nm. NOx concentrations were determined from a linear standard curve established with sodium nitrite (0–100 µM). Sample concentrations were calculated by subtracting the blank absorbance from the sample absorbance and applying the slope and intercept of the standard curve.

Urinary NOx was measured using a Nitric Oxide Assay kit (Oxford Biomedical Research, Inc., Rochester Hills, MI, USA) according to the manufacturer’s instructions. Urine samples were diluted 10-fold with reaction buffer prior to analysis.

### 2.4 Blood glucose measurement

Blood glucose levels were measured from tail-vein blood on days 0, 3, 7, 15, and 30 prior to daily drug administration, using a portable blood glucose monitoring system (i-SENS, Inc., St. Ingbert, Germany). Blood was collected by tail vein puncture and applied directly to a glucose test strip.

### 2.5 Western blot analysis

At the end of the experiment, rats were sacrificed by cervical dislocation, and aortic arch tissue was isolated, dissected, and homogenized in protein extraction solution (Intron Biotechnology, Inc., Seongnam, Korea). Protein concentrations were determined using a BCA assay kit (Thermo Scientific, Waltham, MA, USA). Equal amounts of protein (50 µg) were subjected to SDS-PAGE on a 7.5% gel and transferred to polyvinylidene difluoride (PVDF) membranes (Millipore, Burlington, MA, USA). Membranes were incubated overnight with a primary anti-eNOS antibody (Signalway Antibody, 1:1,000 dilution), followed by the corresponding secondary antibody, and developed using chemiluminescent reagents (Santa Cruz Biotechnology, Dallas, TX, USA). Band intensities were quantified by densitometric analysis using ImageJ software.

### 2.6 LC-MS/MS assay

Plasma concentrations of ARG and CIT were determined by a hydrophilic interaction liquid chromatography-electrospray ionization tandem mass spectrometry (HILIC-ESI-MS/MS) method adapted from previously reported methods (Shin et al., 2011b; Shin et al., 2019). Chromatographic separation was performed on an Alltima HP HILIC column (3 μm, 150 mm × 2.1 mm) maintained at 40 °C using an Agilent 1200 HPLC system. The mobile phase consisted of 0.3% formic acid with 10 mM ammonium formate in water (A) and 0.3% formic acid in acetonitrile (B) at a ratio of 35:65 (v/v, isocratic). The flow rate was 0.4 mL/min, and the injection volume was 3 µL. Total run time was 6 min.

Mass spectrometric detection was performed using an Agilent 6430 triple quadrupole mass spectrometer operating in positive electrospray ionization mode. Multiple reaction monitoring (MRM) transitions were m/z 175.1 → 70.0 for ARG, 176.1 → 70.1 for CIT, 181.1 → 74.2 for ^13^C_6_-ARG, and 180.1 → 74.2 for D_4_-CIT. The dwell time was 200 ms per transition. Gas temperature was set to 350 °C, gas flow to 10 L/min, and nebulizer pressure to 35 psi.

Plasma samples were diluted 20-fold with water prior to preparation. An aliquot of 20 µL of diluted sample was mixed with 40 µL of internal standard solution (^13^C_6_-ARG and D_4_-CIT, each at 1,000 ng/mL) and 40 µL of water, followed by the addition of 400 µL acetonitrile for protein precipitation. After vortexing and centrifugation at 13,200 rpm for 10 min at 4 °C, 200 µL of the supernatant was collected for analysis. Calibration standards were prepared in 4% bovine serum albumin (BSA) in phosphate-buffered saline diluted 20-fold with water, at concentrations of 0.1, 0.5, 1, 5, 10, 25, and 50 μg/mL. Quality control samples were prepared at 0.1, 0.4, 20, and 40 μg/mL. The method was validated in accordance with the FDA guidance for bioanalytical method validation (U.S. FDA, 2018).

### 2.7 Noncompartmental analysis

Noncompartmental analysis (NCA) was performed on the plasma concentration–time profiles. Prior to analysis, plasma concentrations were baseline-corrected by subtracting the individual mean pre-dose concentration, calculated as the average of samples collected at −2, −1, and 0 h, from each observed value. Pharmacokinetic parameters included the apparent elimination half-life (t_1/2_), initial plasma concentration after intravenous administration (C_0_), peak plasma concentration after oral administration (C_max_), time to peak plasma concentration (T_max_), and area under the plasma concentration–time curve from time zero to the last measurable concentration (AUC_last_). The absolute oral bioavailability (F) was calculated as the dose-normalized ratio of AUC_last_ following oral administration to that following intravenous administration.

### 2.8 Population pharmacokinetic modeling

A population pharmacokinetic model was developed to simultaneously characterize the plasma concentration–time profiles of ARG and CIT following intravenous and oral administration using S-ADAPT®. Both ARG and CIT dispositions were described by a two-compartment model. One-compartment and two-compartment structural models were evaluated for both ARG and CIT. The two-compartment model was selected based on the biexponential decline observed in the intravenous concentration–time profiles and a lower objective function value compared with the one-compartment model. To account for endogenous production, zero-order synthesis rates (Syn_ARG_, Syn_CIT_) were derived from baseline plasma concentrations and the respective clearance parameters. A metabolic conversion fraction (F_met_) was incorporated into CIT clearance to represent the fraction of CIT elimination directed toward ARG formation. The model was described by the following differential equations (Equations 1–6):

$$\frac{d{\text{Gut}}_{\mathrm{ARG}}}{\text{dt}}=-k_{a,\mathrm{ARG}}\cdot {\text{Gut}}_{\mathrm{ARG}}$$ (1)

$$\frac{dX_{1,\mathrm{ARG}}}{\text{dt}}=k_{a,\mathrm{ARG}}\cdot {\text{Gut}}_{\mathrm{ARG}}+{\text{Syn}}_{\mathrm{ARG}}+F_{\text{met}}\cdot {\text{CL}}_{\text{CIT}}\cdot C_{1,\text{CIT}}-{\text{CL}}_{\mathrm{ARG}}\cdot C_{1,\mathrm{ARG}}-{\text{CLD}}_{\mathrm{ARG}}\cdot C_{1,\mathrm{ARG}}+{\text{CLD}}_{\mathrm{ARG}}\cdot C_{2,\mathrm{ARG}}$$ (2)

$$\frac{dX_{2,\mathrm{ARG}}}{\text{dt}}={\text{CLD}}_{\mathrm{ARG}}\cdot C_{1,\mathrm{ARG}}-{\text{CLD}}_{\mathrm{ARG}}\cdot C_{2,\mathrm{ARG}}$$ (3)

$$\frac{d{\text{Gut}}_{\text{CIT}}}{\text{dt}}=-k_{a,\text{CIT}}\cdot {\text{Gut}}_{\text{CIT}}$$ (4)

$$\frac{dX_{1,\text{CIT}}}{\text{dt}}=k_{a,\text{CIT}}\cdot {\text{Gut}}_{\text{CIT}}+{\text{Syn}}_{\text{CIT}}-{\text{CL}}_{\text{CIT}}\cdot C_{1,\text{CIT}}-{\text{CLD}}_{\text{CIT}}\cdot C_{1,\text{CIT}}+{\text{CLD}}_{\text{CIT}}\cdot C_{2,\text{CIT}}$$ (5)

$$\frac{dX_{2,\text{CIT}}}{\text{dt}}={\text{CLD}}_{\text{CIT}}\cdot C_{1,\text{CIT}}-{\text{CLD}}_{\text{CIT}}\cdot C_{2,\text{CIT}}$$ (6)

where Gut_ARG_ and Gut_CIT_ are the amounts of ARG and CIT in the gut compartment; X_1,ARG_, X_2,ARG_, X_1,CIT_, and X_2,CIT_ are the amounts of ARG and CIT in the central and peripheral compartments; C_1,ARG_ = X_1,ARG_/V_1,ARG_, C_2,ARG_ = X_2,ARG_/V_2,ARG_, C_1,CIT_ = X_1,CIT_/V_1,CIT_, and C_2,CIT_ = X_2,CIT_/V_2,CIT_ are the corresponding plasma concentrations; V_1,ARG_ and V_1,CIT_ are the central volumes of distribution of ARG and CIT, and V_2,ARG_ and V_2,CIT_ are the peripheral volumes of distribution of ARG and CIT; k_a,ARG_ and k_a,CIT_ are the first-order absorption rate constants; CL_ARG_ and CL_CIT_ are the systemic clearances; CLD_ARG_ and CLD_CIT_ are the distributional clearances; and Syn_ARG_ and Syn_CIT_ are the zero-order endogenous synthesis rates, derived from baseline plasma concentrations (B_ARG_ and B_CIT_) and clearance parameters as (Equations 7–8):

$${\text{Syn}}_{\mathrm{ARG}}=B_{\mathrm{ARG}}\cdot {\text{CL}}_{\mathrm{ARG}}-F_{\text{met}}\cdot {\text{CL}}_{\text{CIT}}\cdot B_{\text{CIT}}$$ (7)

$${\text{Syn}}_{\text{CIT}}=B_{\text{CIT}}\cdot {\text{CL}}_{\text{CIT}}$$ (8)

Nonlinear mixed-effects modeling was used to estimate population mean parameters and between-subject variability (BSV), with BSV modeled using an exponential error model**.** Oral bioavailability parameters (F_ARG_, F_CIT_) and the metabolic conversion fraction (F_met_) were modeled using a logit transformation to constrain estimates within the physiological range of 0–1. A combined additive and proportional error model was used to describe residual variability.

### 2.9 Statistical analysis

All data are expressed as the mean ± SD. Statistical significance was determined using one-way analysis of variance (one-way ANOVA) with Tukey’s *post hoc* test for comparisons among groups. For blood glucose levels measured repeatedly over time, two-way ANOVA (mixed-effects analysis) with Tukey’s multiple comparisons test was used to assess the effects of treatment and time. A p-value of less than 0.05 was considered statistically significant.

## 3 Results

### 3.1 Plasma and urinary NOx levels

**FIGURE 1:**
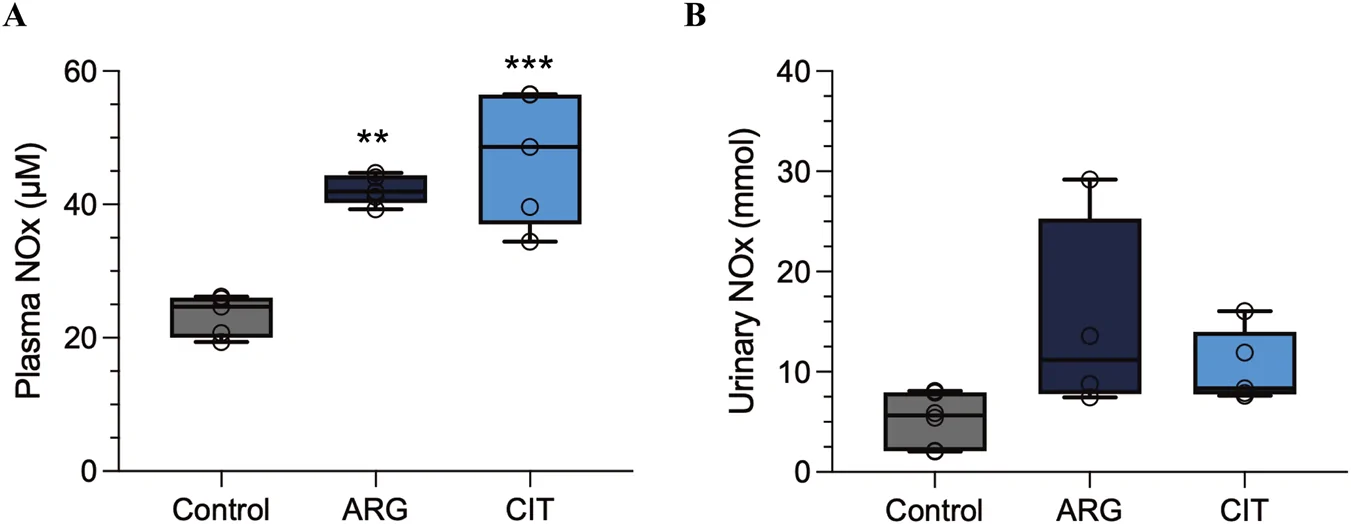
NOx concentrations in **(A)** plasma and **(B)** urine in rats following 30-day oral supplementation with L-alanine as control, ARG, or CIT (500 mg/kg/day, n = 6 each). NOx (nitrite and nitrate) was measured at day 30. Data are expressed as median with interquartile range; individual data points are shown. Statistical significance was assessed by one-way ANOVA with Tukey’s *post hoc* test. **, p < 0.01, ***p < 0.001 vs. control.

All animals remained healthy throughout the 30-day treatment period of oral ARG or CIT with no signs of overt toxicity. Body mass did not differ significantly among groups at any time point during the study, suggesting that the supplements did not affect overall growth. At day 30, plasma NOx levels were approximately two-fold higher in ARG- and CIT-treated rats than in controls (p < 0.05, Figure 1A), indicating enhanced systemic NO production following supplementation with either compound. Urinary NOx excretion tended to be higher in both treatment groups compared to controls, with total NOx excretion approximately three-fold and two-fold higher in ARG- and CIT-treated rats, respectively, although this difference did not reach statistical significance (p = 0.066, Figure 1B).

### 3.2 eNOS protein expression

**FIGURE 2:**
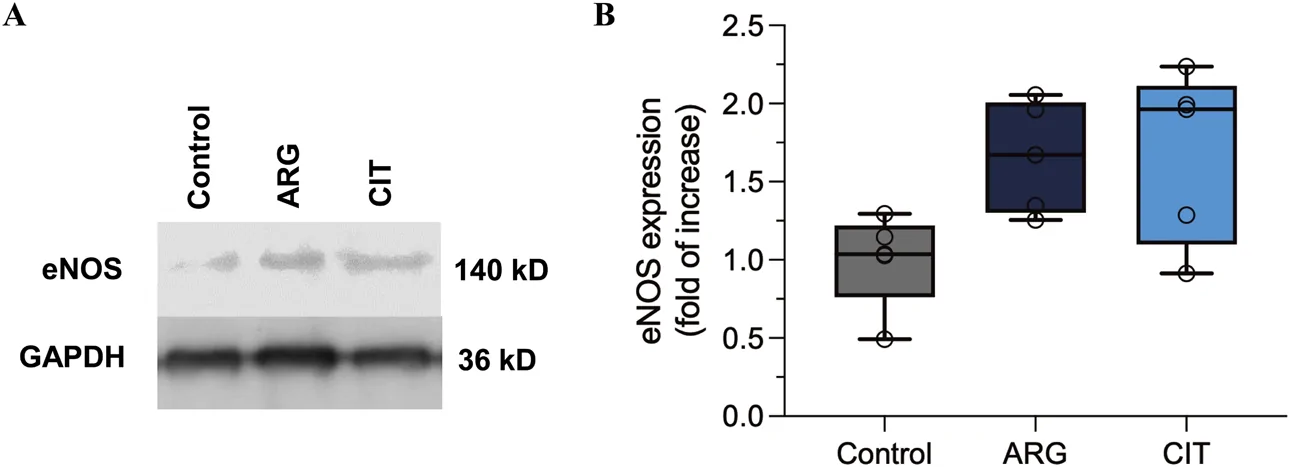
eNOS protein expression in aortic arch tissue following 30-day oral supplementation with L-alanine as control, ARG, or CIT (500 mg/kg/day, n = 6 each) **(A)** Representative Western blot images of eNOS (140 kDa) and GAPDH (36 kDa) **(B)** Densitometric quantification of eNOS normalized to the GAPDH loading control, expressed as fold increase relative to control. Data are expressed as median with interquartile range; individual data points are shown.

Figure 2 shows Western blot analysis of aortic arch tissue following 30-day oral administration of ARG and CIT. Higher eNOS protein levels were observed in both the ARG and CIT groups compared with the control group. Immunoreactive bands at 140 kDa were more intense in ARG- and CIT-treated rats than in controls (Figure 2A). Densitometric analysis showed that eNOS protein expression was approximately 1.6-fold higher in both ARG and CIT groups compared to controls (Figure 2B), with one-way ANOVA indicating overall significance (p < 0.05); however, pairwise differences between groups did not reach statistical significance in *post hoc* analysis.

### 3.3 Blood glucose levels

**FIGURE 3:**
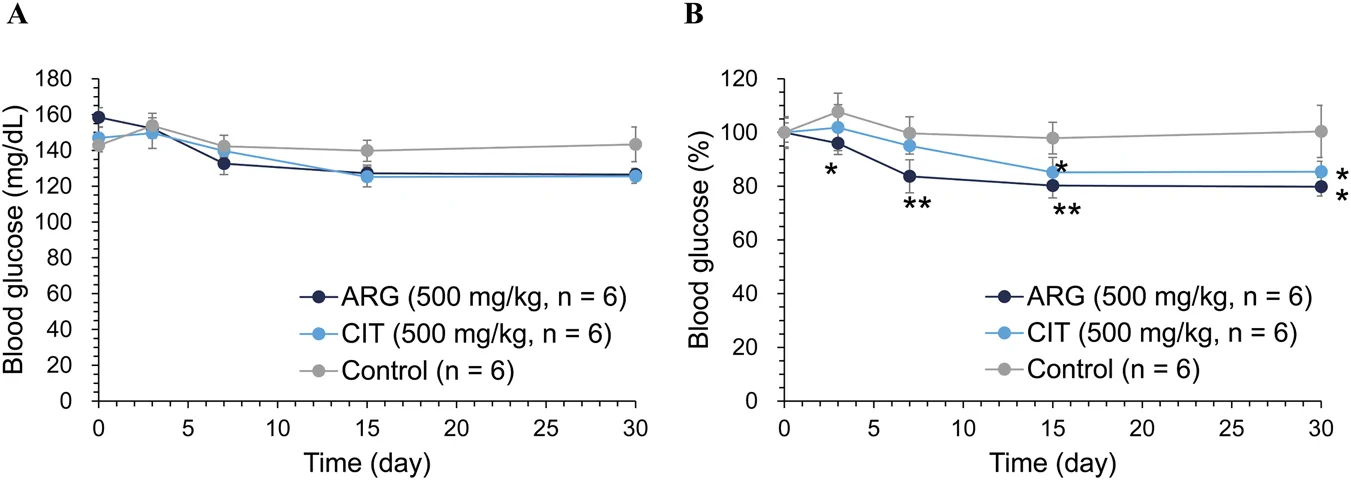
Blood glucose levels in rats during 30-day oral supplementation with ARG, CIT (500 mg/kg/day, n = 6 each), or L-alanine as a control (n = 6), expressed as **(A)** absolute concentrations and **(B)** percentage of day 0 values. Blood glucose was measured on days 0, 3, 7, 15, and 30 prior to daily administration; day 0 represents the baseline value before supplementation. Data are expressed as mean ± SD. A significant effect of treatment was observed by two-way ANOVA (mixed-effect analysis) with Tukey’s multiple comparisons test. *p < 0.05, **p < 0.01 vs. control.

Blood glucose levels showed no statistically significant difference among groups at baseline (Day 0, before the initiation of supplementation) and remained stable in the control group throughout the study period (Figure 3A). In contrast, blood glucose levels progressively decreased in both ARG and CIT groups from day 3 onward. When expressed as a percentage of baseline, this reduction was statistically significant in the ARG group from day 3, and in both ARG and CIT groups from day 15 (p < 0.05; Figure 3B).

### 3.4 Pharmacokinetics of ARG and CIT

**FIGURE 4:**
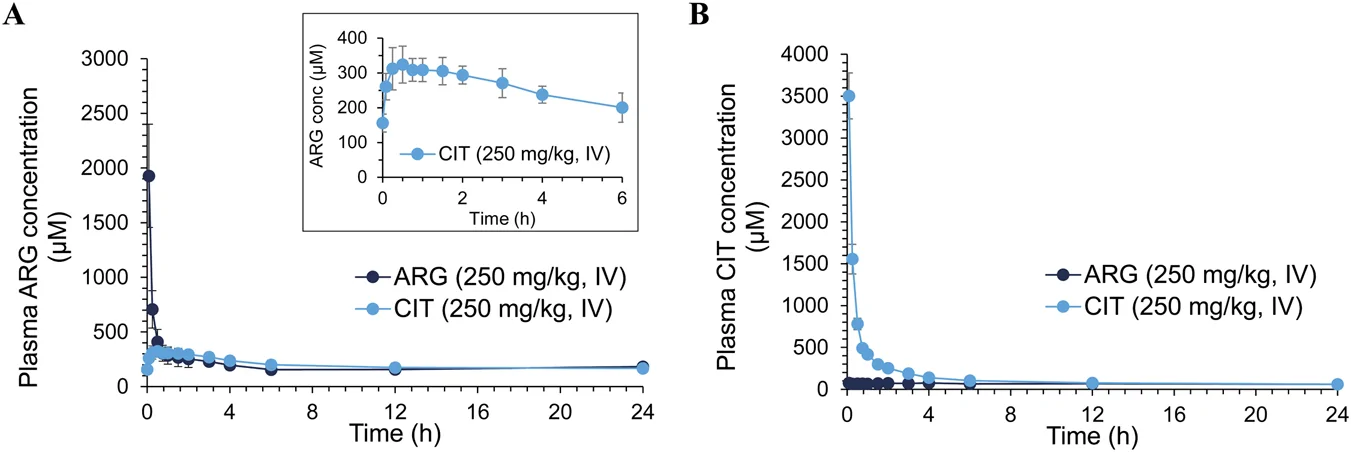
Plasma concentration–time profiles of **(A)** ARG and **(B)** CIT following intravenous (IV) injection of ARG or CIT (250 mg/kg, n = 4 per group). Both ARG and CIT concentrations were measured following administration of either compound. Data are expressed as mean ± SD of measured (uncorrected) concentrations. Inset in **(A)** shows plasma ARG concentrations following CIT IV injection on an expanded time scale (0–6 h).

**FIGURE 5:**
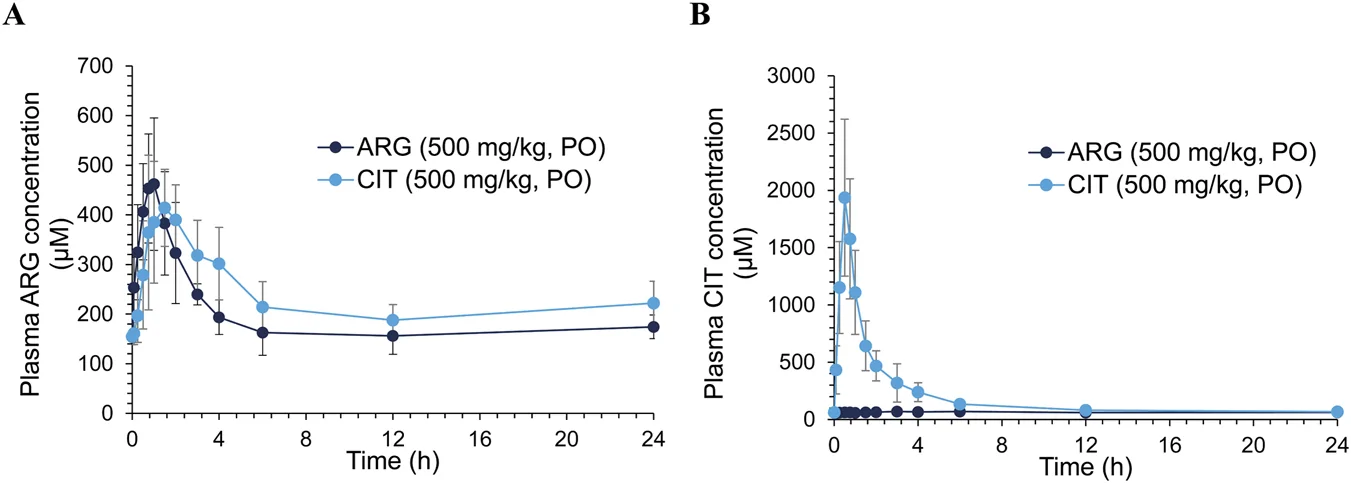
Plasma concentration–time profiles of **(A)** ARG and **(B)** CIT following oral (PO) administration of ARG or CIT (500 mg/kg, n = five to six per group). Both ARG and CIT concentrations were measured following administration of either compound. Data are expressed as mean ± SD of measured (uncorrected) concentrations.

Plasma concentration–time profiles of ARG and CIT following intravenous and oral administration are shown in Figures 4, 5. Both ARG and CIT were quantified simultaneously following administration of either compound. Table 1 summarizes noncompartmental pharmacokinetic parameters derived from baseline-corrected concentrations; accordingly, all parameter estimates may differ from values apparent in the figures, which display uncorrected measured plasma concentrations.

**TABLE 1:** Noncompartmental pharmacokinetic parameters of L-arginine (ARG) and L-citrulline (CIT) following intravenous (IV) and oral (PO) administration in rats (mean ± SD).

Route (dose)	Administration	ARG administration	CIT administration	
	Measured	ARG	ARG	CIT
IV (250 mg/kg)	t_1/2_ (h)	1.14 ± 0.36	3.20 ± 1.63*	3.24 ± 1.04
	T_max_ (h)	–	0.625 (0.5, 2.0)	–
	C_0_ or C_max_ (μM)	3227.98 ± 1039.51	157.06 ± 28.62	5273.89 ± 853.61
	AUC_last_ (μM·h)	1202.93 ± 584.05	904.73 ± 224.07	2050.78 ± 82.65
	AUC_inf_ (μM·h)	1244.14 ± 607.23	1000.45 ± 221.39	2085.14 ± 97.31
	CL (L·h^-1^·kg^-1^)	1.32 ± 0.47	–	0.69 ± 0.03
	V_ss_ (L/kg)	6.43 ± 5.11	–	1.22 ± 0.31
PO (500 mg/kg)	t_1/2_ (h)	1.91 ± 2.45	5.25 ± 4.60	3.88 ± 2.51
	T_max_ (h)	1.0 (0.25, 1.0)	1.0 (0.75, 1.50)	0.50 (0.50, 0.75)
	C_max_ (μM)	333.29 ± 111.12	281.21 ± 97.16	1902.33 ± 677.90
	AUC_last_ (μM·h)	1260.63 ± 536.66	2013.94 ± 888.13	3186.77 ± 1106.86
	AUC_inf_ (μM·h)	1404.80 ± 787.94	2433.05 ± 1296.92	3236.81 ± 1121.42
	Bioavailability	0.52	–	0.78

All pharmacokinetic parameters were derived from baseline-corrected plasma concentrations. T_max_ is expressed as median (minimum, maximum); all other parameters are expressed as mean ± SD. –, not available.

* p < 0.05 vs. ARG administration.

Following intravenous ARG injection (250 mg/kg), ARG concentrations peaked immediately and declined rapidly (Figure 4A). Following intravenous CIT injection, ARG concentrations also rose promptly, reaching a peak of 157.06 ± 28.62 μM at 0.625 h (Figure 4A inset; Table 1), demonstrating rapid *in vivo* conversion of CIT to ARG. The apparent half-life of ARG after CIT administration (3.20 ± 1.63 h) was significantly longer than that after direct ARG administration (1.14 ± 0.36 h; p < 0.05), and closely matched the half-life of CIT itself (3.24 ± 1.04 h). This likely reflects flip-flop kinetics, whereby the terminal half-life of plasma ARG is governed by the slower elimination of CIT rather than by intrinsic ARG elimination. Meanwhile, CIT concentrations following intravenous CIT injection peaked at 5273.89 ± 853.61 µM and declined with a half-life of 3.24 ± 1.04 h (Figure 4B; Table 1). CIT concentrations were unaffected by ARG intravenous injection (Figure 4B), indicating that the conversion is unidirectional.

After oral administration (500 mg/kg), both compounds were rapidly absorbed with T_max_ within 1 h (Table 1; Figure 5). CIT was absorbed more rapidly than ARG, as indicated by a shorter T_max_ (0.50 vs. 1.0 h). Following oral ARG administration, ARG concentrations peaked at 333.29 ± 111.12 µM (Figure 5A; Table 1). Following oral CIT administration, the resulting ARG C_max_ (281.21 ± 97.16 µM) was comparable to that following direct oral ARG administration, while the AUC_last_ of ARG tended to be higher (2013.94 ± 888.13 vs. 1260.63 ± 536.66 μM·h), although the difference was not statistically significant. CIT showed substantially greater systemic exposure than ARG after oral administration, as reflected by markedly higher C_max_ and AUC_last_ values (Table 1). The apparent half-life of ARG after oral CIT administration (5.25 ± 4.60 h) was also longer than after direct ARG administration (1.91 ± 2.45 h), consistent with flip-flop kinetics as observed following intravenous administration. The oral bioavailability of CIT (78%) was substantially higher than that of ARG (52%).

### 3.5 Population pharmacokinetic modeling

**FIGURE 6:**
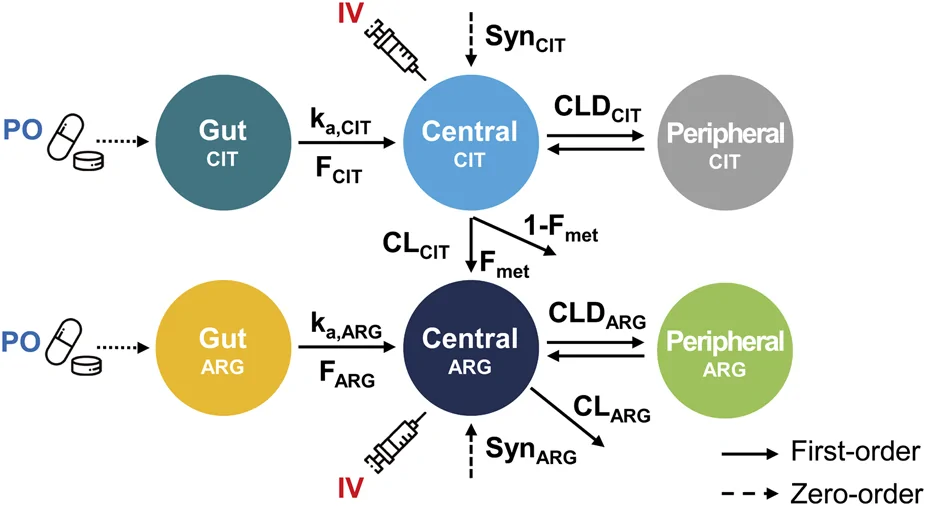
Schematic representation of the population pharmacokinetic model for ARG and CIT. ARG and CIT dispositions were both described by a two-compartment model, linked via fractional conversion of CIT clearance to ARG (F_met_). Endogenous synthesis was incorporated as zero-order input rates (Syn_ARG_, Syn_CIT_). Oral (PO) doses enter through the gut compartment, whereas intravenous (IV) doses enter the central compartment directly. k_a_, absorption rate constant; F, oral bioavailability; CL, clearance; CLD, distributional clearance.

A population pharmacokinetic model linking ARG and CIT disposition was developed to provide a quantitative framework for interpreting the pharmacodynamic effects observed with both supplements (Figure 6).

**FIGURE 7:**
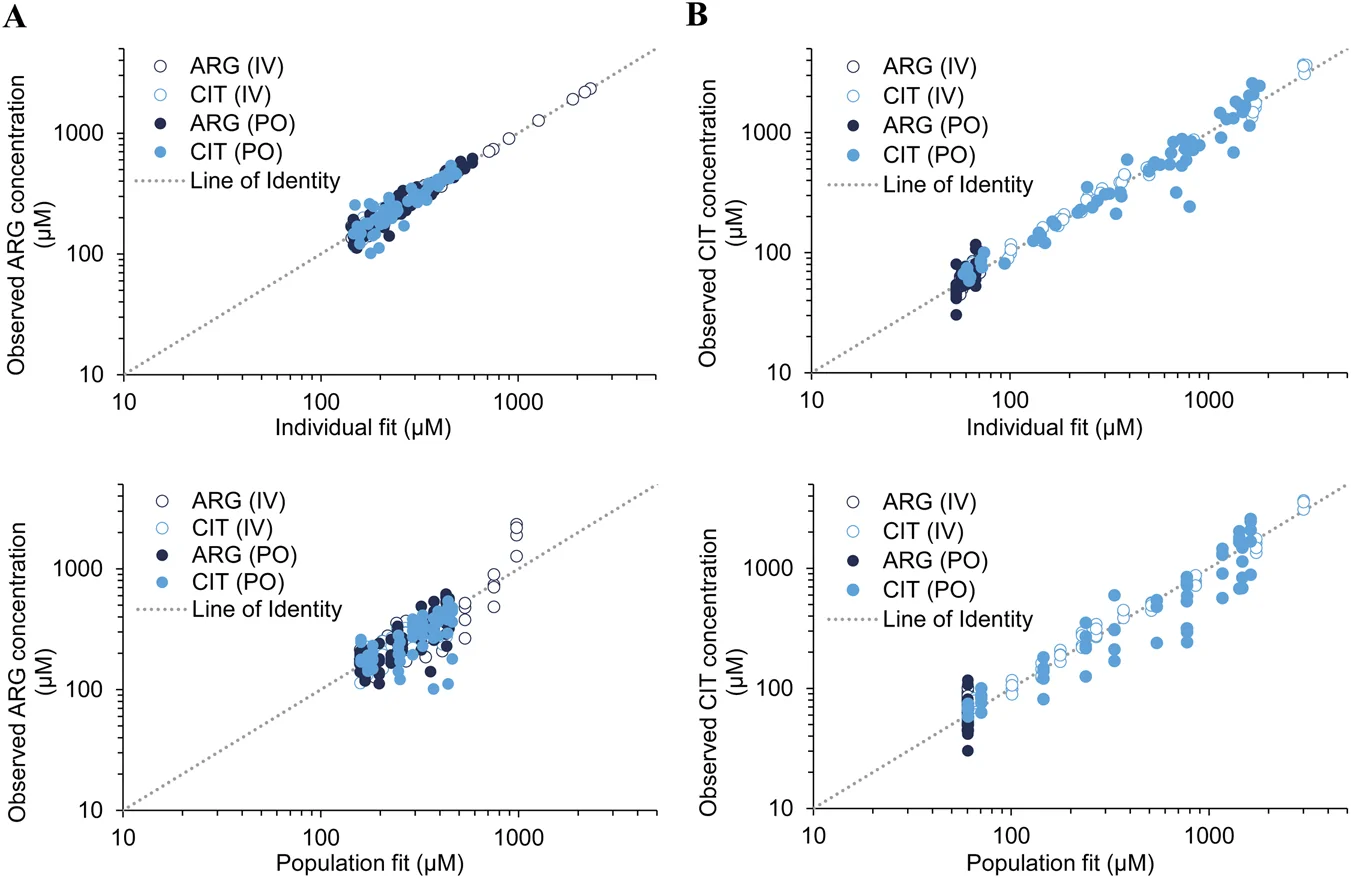
Goodness-of-fit plots for the population pharmacokinetic model. Observed plasma **(A)** ARG and **(B)** CIT concentrations are plotted against individual (top) and population (bottom) model fits. The dotted line represents the line of identity. Symbols represent the administered compound and route of administration: ARG (IV), CIT (IV), ARG (PO), and CIT (PO). IV, intravenous; PO, oral.

**FIGURE 8:**
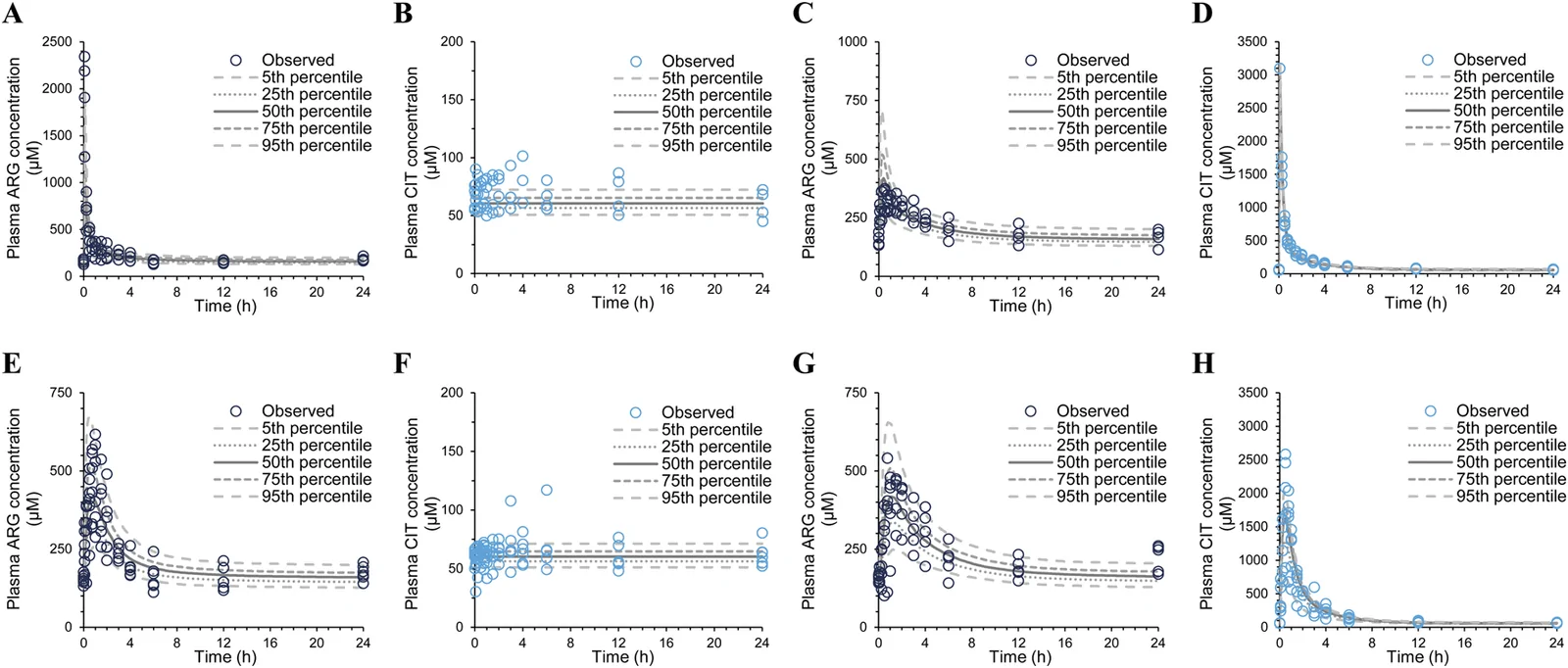
Visual predictive checks for the population pharmacokinetic model, stratified by analyte, administered compound, and route of administration. Observed plasma concentrations (open circles) are overlaid with the fifth, 25th, 50th, 75th, and 95th percentiles predicted from 1000 simulated datasets (lines). Each panel shows the concentration of one analyte following administration of one compound by one route **(A)** ARG after ARG IV **(B)** CIT after ARG IV **(C)** ARG after CIT IV **(D)** CIT after CIT IV **(E)** ARG after ARG PO **(F)** CIT after ARG PO **(G)** ARG after CIT PO **(H)** CIT after CIT PO. IV, intravenous; PO, oral.

A two-compartment model with first-order absorption and first-order elimination from the central compartment was used to describe the disposition of both ARG and CIT. Zero-order endogenous synthesis (Syn_ARG_, Syn_CIT_) was incorporated as a baseline input, and a metabolic conversion fraction (F_met_) was included to represent the fraction of CIT clearance directed toward ARG formation. The model simultaneously characterized plasma ARG and CIT concentrations following intravenous and oral administration of both compounds within a single framework. Goodness-of-fit plots demonstrated close agreement between observed and predicted concentrations for both ARG and CIT (Figure 7), and visual predictive checks confirmed that the model adequately captured the central tendency and variability of the observed concentration–time profiles across all routes of administration (Figure 8).

Parameter estimates for the final model are summarized in Table 2, and all parameters were estimated with acceptable precision. The model-estimated oral bioavailability was substantially higher for CIT than that for ARG (F_CIT_, 82.2% vs. F_ARG_, 52.6%), consistent with the NCA-derived estimates (78% vs. 53%, respectively). The model-estimated absorption rate constant was higher for CIT than that for ARG (k_a, CIT_, 1.63 h^-1^ vs. k_a, ARG_ = 1.17 h^-1^), suggesting more rapid absorption of CIT. The model-estimated F_met_ was close to unity (0.999), indicating that CIT elimination was predominantly directed toward ARG formation. This suggests that CIT administration may provide systemic ARG exposure comparable to or greater than that following direct oral ARG administration. Endogenous synthesis rates were estimated as Syn_ARG_ = 210.3 μmol·h^-1^·kg^-1^ and Syn_CIT_ = 45.7 μmol·h^-1^·kg^-1^, derived from the model-estimated baseline plasma concentrations and clearance parameters at steady-state conditions.

**TABLE 2:** Population pharmacokinetic parameter estimates for ARG and CIT.

Parameter	Symbol	Unit	Population mean (SE%)	BSV (SE%)
Absorption rate constant of ARG	k_a, ARG_	h^-1^	1.17 (9%)	0.00512 (234%)
Absorption rate constant of CIT	k_a, CIT_	h^-1^	1.63 (9.2%)	0.00636 (188%)
Oral bioavailability of ARG	F_ARG_	–	0.526 (377%)	0.226 (124%)
Oral bioavailability of CIT	F_CIT_	–	0.822 (44.4%)	1.82 (74.3%)
Fraction of CIT clearance converted to ARG	F_met_	–	0.999 (15.7%)	4.61 (63.3%)
Systemic clearance of ARG	CL_ARG_	L·h^-1^·kg^-1^	1.62 (12.7%)	0.0166 (261%)
Systemic clearance of CIT	CL_CIT_	L·h^-1^·kg^-1^	0.757 (4.8%)	0.00046 (194%)
Central volume of distribution of ARG	V_1, ARG_	L·kg^-1^	1.49 (21.8%)	0.773 (33.6%)
Peripheral volume of distribution of ARG	V_2, ARG_	L·kg^-1^	2.89 (67.8%)	1.48 (59%)
Central volume of distribution of CIT	V_1, CIT_	L·kg^-1^	0.359 (7.8%)	0.0105 (101%)
Peripheral volume of distribution of CIT	V_2, CIT_	L·kg^-1^	0.824 (5.8%)	0.00272 (190%)
Distributional clearance of ARG	CLD_ARG_	L·h^-1^·kg^-1^	1.39 (35.9%)	0.804 (77.3%)
Distributional clearance of CIT	CLD_CIT_	L·h^-1^·kg^-1^	0.54 (25.6%)	0.0339 (351%)
Baseline plasma concentration of ARG	B_ARG_	µM	158 (4.2%)	0.0184 (59.1%)
Baseline plasma concentration of CIT	B_CIT_	µM	60.4 (3.9%)	0.0109 (87.1%)

## 4 Discussion

This study provides quantitative pharmacokinetic evidence that oral CIT functions as an effective ARG precursor *in vivo*. Although the CIT-to-ARG conversion is well established, its quantitative contribution to systemic ARG exposure and NO-mediated pharmacodynamic effects has not been previously characterized using a pharmacokinetic modeling framework. To address this, long-term pharmacodynamic effects of ARG and CIT supplementation were assessed over 30 days, and a population PK model was developed from single-dose intravenous and oral data to provide a mechanistic basis for the observed outcomes. The model estimated a high oral bioavailability of CIT and suggested that CIT elimination was predominantly attributable to conversion to ARG, thereby providing a quantitative explanation for the similar pharmacodynamic efficacy observed with both supplements.

Both ARG and CIT supplementation increased plasma NOx levels, upregulated eNOS expression, and reduced blood glucose, consistent with activation of the ARG–NO pathway (Schwedhelm et al., 2008; Claybaugh et al., 2014; Forzano et al., 2023; Park et al., 2023). These findings are consistent with previous reports showing that exogenous ARG can enhance NO production *in vivo* despite intracellular ARG concentrations theoretically saturating eNOS, i.e., a phenomenon known as the L-arginine paradox. Baseline plasma ARG concentrations estimated in the present model (B_ARG_ = 158 µM) substantially exceed the K_m_ of eNOS, confirming the relevance of this paradox in the present experimental context, and supporting the relevance of pharmacokinetic factors governing ARG availability in the interpretation of NO-mediated outcomes (Tsikas et al., 2000; Boger, 2004; Shin et al., 2011c; Shin et al., 2017).

The model-estimated oral bioavailability of ARG (F_ARG_ = 52.6%) is consistent with the well-established first-pass extraction of ARG by intestinal and hepatic arginase (Tangphao et al., 1999; Schwedhelm et al., 2008). In contrast, CIT demonstrated substantially higher oral bioavailability (F_CIT_ = 82.2%), consistent with its known resistance to arginase-mediated first-pass extraction (Allerton et al., 2018; Papadia et al., 2018). Both estimates were in close agreement with NCA-derived values (53% and 78%, respectively), supporting the internal consistency of the model. The high model-estimated F_met_ indicates that conversion to ARG is the predominant elimination pathway for CIT. This was corroborated by independent NCA-based estimates (approximately 0.81–0.93; Supplementary Table S1) (Kaplan et al., 1973; Rowland and Tozer, 2011; Shin B. S. et al., 2011). A sensitivity analysis further indicated that only a high conversion fraction could reproduce the observed ARG concentrations, as lower F_met_ values systematically underpredicted plasma ARG (Supplementary Figure S1). In addition, F_met_ was not strongly correlated with the other model parameters. Mechanistically, CIT undergoes minimal renal excretion and is preferentially converted to ARG in the kidneys via argininosuccinate synthetase and argininosuccinate lyase, supporting this estimate (Agarwal et al., 2017). Combined with high oral bioavailability, oral CIT produced numerically higher AUC_last_ of ARG than direct oral ARG administration (2013.94 ± 888.13 vs. 1260.63 ± 536.66 μM·h), although the difference was not statistically significant. Furthermore, the prolonged apparent half-life of ARG following CIT administration reflects flip-flop kinetics, in which the slower elimination of CIT sustains renal conversion, thereby prolonging ARG supply. These findings provide a quantitative mechanistic explanation for the efficacy of oral CIT as an ARG precursor, consistent with previous reports in humans and animal models (Osowska et al., 2004; Moinard et al., 2008; Marini et al., 2010).

A notable strength of the present study is the integrated experimental and modeling design. ARG and CIT were quantified simultaneously following administration of either compound across both intravenous and oral routes, and all concentration–time profiles were characterized within a single population PK framework. This design enabled direct characterization of the asymmetric CIT-to-ARG conversion *in vivo*, i.e., ARG concentrations rose promptly after intravenous CIT injection, while CIT concentrations were unaffected by ARG administration (Figures 4, 5). This integrated approach also allowed endogenous baseline contributions of both compounds to be explicitly accounted for. While most preclinical studies have examined ARG or CIT over short periods, the present study is among the few to directly integrate long-term pharmacodynamic outcomes with a quantitative population PK characterization in the same experimental system (Rashid et al., 2020).

CIT demonstrated a more favorable pharmacokinetic profile compared to ARG, including higher oral bioavailability, near-complete conversion to ARG, and more sustained systemic ARG supply. Nevertheless, pharmacodynamic responses were not significantly different between the two groups. Therefore, the advantages of CIT identified in this study are pharmacokinetic rather than pharmacodynamic in nature. This suggests that systemic ARG exposure with either supplement was sufficient to sustain the ARG–NO pathway, and that further increases in ARG exposure did not translate into additional pharmacodynamic effects under the present experimental conditions. These findings are consistent with a plateau relationship between systemic ARG exposure and downstream NO-mediated outcomes, rather than a simple linear association. However, long-term ARG supplementation has been associated with upregulation of arginase-II activity, eNOS uncoupling, and reduced sustained efficacy (Xiong et al., 2014; Allerton et al., 2018). By comparison, CIT is not a substrate for arginase, and no adverse effects of long-term CIT supplementation have been reported (Papadia et al., 2018; Gonzalez et al., 2023). No signs of overt toxicity were observed in either group over the 30-day supplementation period, further supporting the potential advantage of CIT over direct ARG supplementation from a pharmacokinetic perspective.

Nevertheless, several limitations of the present study warrant consideration. The use of a rat model limits direct extrapolation to human physiology, where differences in renal CIT-to-ARG conversion efficiency and first-pass ARG metabolism may alter the quantitative relationships described here. In addition, the relatively small intravenous group sizes may have contributed to uncertainty in some parameter estimates. Whether the estimated F_met_ remains constant across a range of CIT doses requires further investigation, and the plasma-only design may limit its precise estimation. As ARG and CIT are endogenous compounds, baseline correction and associated model assumptions may have influenced parameter estimation. Finally, the model does not account for tissue-level differences in ARG availability at the site of eNOS activity, which may be an important determinant of NO production independent of systemic exposure. Future studies incorporating isotopically labeled CIT could experimentally validate the CIT-to-ARG conversion and the associated pharmacokinetic parameters.

## 5 Conclusions

In conclusion, this study provides a quantitative pharmacokinetic basis for the NO-mediated effects of oral ARG and CIT supplementation. The high oral bioavailability of CIT and its predominant *in vivo* conversion to ARG, as quantified by population PK modeling, support CIT as an effective oral ARG precursor. While the pharmacodynamic effects of CIT and ARG were comparable, CIT offers a more favorable pharmacokinetic profile, supporting its potential as a pharmacokinetically advantageous alternative to oral ARG supplementation.

## Data Availability

The original contributions presented in the study are included in the article/Supplementary Material, further inquiries can be directed to the corresponding authors.
