# Supplementary material for: Quantitative characterization of L-citrulline as a precursor of L-arginine in rats: pharmacokinetic basis for nitric oxide-mediated effects
Source: Front Pharmacol. 2026 Sep 3;17:1889835. doi: 10.3389/fphar.2026.1889835 (PMC13581722; doi:10.3389/fphar.2026.1889835)

Supplementary Table S1. Estimation of the metabolic conversion fraction (F_{met}) of CIT to ARG using three model-independent non-compartmental methods.

Equation	F_{met}	Reference
$F_{met} = \frac{AUC_{ARG\ after\ CIT\ IV\ injection}}{AUC_{ARG\ after\ ARG\ IV\ injection}} \times \frac{Dose_{mol,ARG}}{Dose_{mol,CIT}}$	0.809	(Kaplan et al., 1973; Shin et al., 2011)
$F_{met} = \frac{AUC_{ARG\ after\ CIT\ IV\ injection}}{AUC_{CIT\ after\ CIT\ IV\ injection}} \times \frac{CL_{ARG}}{CL_{CIT}}$	0.926	(Rowland and Tozer, 2011)
$F_{met} = \frac{CL_{ARG}}{CL_{ARG,apparent\ after\ CIT\ IV\ injection}}$	0.887	(Rowland and Tozer, 2011)

F_{met} was estimated using baseline-subtracted concentrations; AUC and CL values were obtained from the non-compartmental analysis (Table 1). $CL_{ARG,\ apparent\ after\ CIT\ IV\ injection}$ denotes the CIT dose divided by the AUC of ARG after CIT IV injection.

References

Kaplan, S.A., Jack, M.L., Cotler, S., and Alexander, K. (1973). Utilization of area under the curve to elucidate the disposition of an extensively biotransformed drug. *Journal of Pharmacokinetics and Biopharmaceutics* 1(3), 201-216. doi: 10.1007/BF01062347.

Rowland, M., and Tozer, T.N. (2011). *Clinical Pharmacokinetics and Pharmacodynamics: Concepts and Applications.* Philadelphia: Lippincott Williams & Wilkins.

Shin, B.S., Bulitta, J.B., Hong, D.K., Kim, H.Y., Kim, M.K., Choi, Y., et al. (2011). Population pharmacokinetics of a novel histone deacetylase inhibitor, cyclo(2S)-2-amino-8-[(aminocarbonyl)hydrazono] decanoyl-1-L-tryptophyl-L-isoleucyl-(2R)-2-piperidinecarbonyl (SD-2007), and its metabolic conversion to apicidin after intravenous injection and oral administration in rats. *Chemotherapy* 57(3), 259-267. doi: 10.1159/000328027.

Supplementary Figure S1. Sensitivity analysis evaluating the impact of key model parameters (F_{met}, CL_{ARG}, and CL_{CIT}) on plasma ARG concentrations following CIT IV administration. The left panels present the simulated plasma ARG concentration–time profiles compared with observed data (open circles). The middle and right panels depict the relative percentage changes (%) in baseline-subtracted (net) AUC_{24h} and C_{max}, respectively, relative to the reference model.

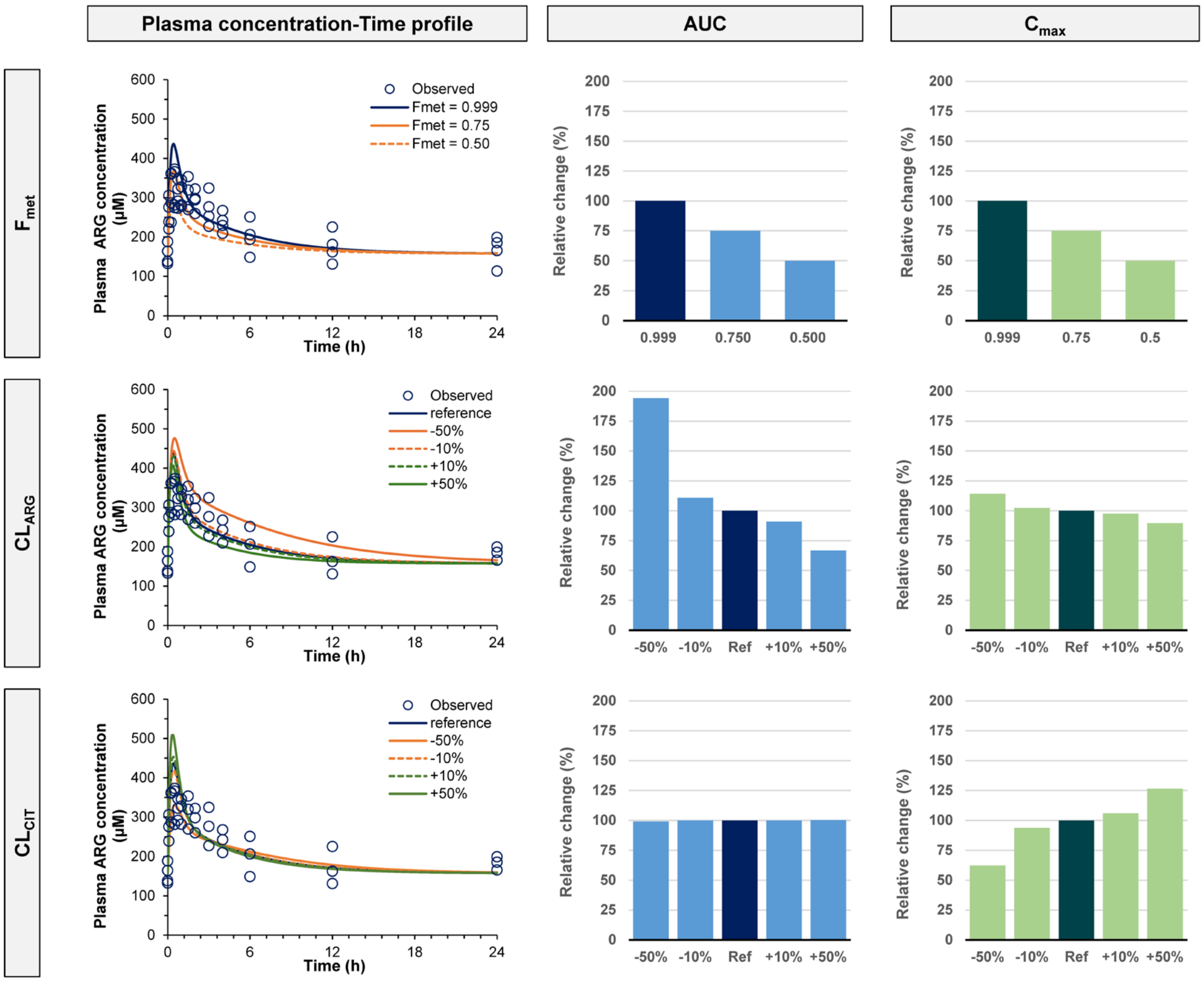

Supplement: Supplementary file 1 [file DataSheet1.pdf]
